# Analysis of the expression pattern of the *BCL11B *gene and its relatives in patients with T-cell acute lymphoblastic leukemia

**DOI:** 10.1186/1756-8722-3-44

**Published:** 2010-11-16

**Authors:** Xin Huang, Shaohua Chen, Qi Shen, Lijian Yang, Bo Li, Liye Zhong, Suxia Geng, Xin Du, Yangqiu Li

**Affiliations:** 1 Institute of Hematology, Medical College, Jinan University, Guangzhou, 510632, PR China; 2 Department of Hematology, Guangdong General Hospital (Guangdong Academy of Medical Sciences), Guangzhou, 510080, PR China; 3 Key Laboratory for Regenerative Medicine of Ministry of Education, Jinan University, Guangzhou, 510632, PR China

## Abstract

**Background:**

In a human T-cell acute lymphoblastic leukemia (T-ALL) cell line (Molt-4), siRNA-mediated suppression of *BCL11B *expression was shown to inhibit proliferation and induce apoptosis, functions which may be related to genes involved in apoptosis (such as *TNFSF10 *and *BCL2L1*) and TGF-β pathways (such as *SPP1*and *CREBBP*).

**Methods:**

The expression levels of the above mentioned genes and their correlation with the *BCL11B *gene were analyzed in patients with T-ALL using the TaqMan and SYBR Green I real-time polymerase chain reaction technique.

**Results:**

Expression levels of *BCL11B, BCL2L1*, and *CREBBP *mRNA in T-ALL patients were significantly higher than those from healthy controls (*P <*0.05). In T-ALL patients, the *BCL11B *expression level was negatively correlated with the *BCL2L1 *expression level (*r*_s _= -0.700; *P <*0.05), and positively correlated with the *SPP1 *expression level (*r*_s _= 0.683; *P <*0.05). In healthy controls, the *BCL11B *expression level did not correlate with the *TNFSF10*, *BCL2L1*, *SPP1*, or *CREBBP *expression levels.

**Conclusions:**

Over-expression of *BCL11B *might play a role in anti-apoptosis in T-ALL cells through up-regulation of its downstream genes *BCL2L1 *and *CREBBP*.

## Background

T-cell acute lymphoblastic leukemia (T-ALL) accounts for 15% of newly diagnosed ALL cases in children and 20-25% of ALL cases in adults [1,2]. Overall, these are aggressive malignancies that do not respond well to chemotherapy and have a poorer prognosis than their B-cell counterparts [3]. The development of targeted therapies, including monoclonal antibodies and gene therapy, continues. Small interfering RNA (siRNA) is a promising gene-targeting agent that has shown great potential, particularly in the field of cancer treatment [4-6].

The B-cell chronic lymphocytic leukemia (CLL)/lymphoma 11B (*BCL11B*) gene plays a crucial role in T-cell development, differentiation, and proliferation [7], and altered expression, mutation, disruption, or rearrangement of *BCL11B *have been associated with T-cell malignancies [8-11]. *BCL11B *over-expression has been observed primarily in T-cell malignancies [8,12]. *BCL11B *has been hypothesized to act as a tumor suppressor gene [9,13], but its precise function remains unclear.

BCL2-like 1 (*BCL2L1; Bcl-xL*) is similar to Bcl-2 because it restrains the apoptosis induction of multiple stimuli, and is a key factor in the terminal step of apoptosis regulation. Studies have shown that *BCL2L1 *participates in various protein-protein interactions, playing a role in inhibiting apoptosis. In the endogenous apoptosis pathway, *BCL2L1 *of the BCL-2 family inhibits apoptosis by blocking the translocation of Bax to the mitochondrial outer membrane [14]. cAMP-response element binding protein (*CREBBP*) plays a critical role in embryonic development, growth control, and homeostasis by coupling chromatin remodeling to transcription factor recognition. A *CREBBP *gene rearrangement with chromosomal translocation has been identified in acute myeloid leukemia [15,16] and over-expression of CREBBP was found in Jurkat cells. Additionally, enhancement of apoptotic cell death occurred in the presence of CREB1 siRNA [17]. Tumor necrosis factor (ligand) superfamily, member 10 (*TNFSF10; TRAIL*) is a tumor necrosis factor superfamily member, and induces apoptosis through its interaction with death receptors. BCL-2 family genes and *TNFSF10 *probably act together through crosstalk between the intrinsic and death receptor-mediated apoptosis pathways [18]. Secreted phosphoprotein 1 (*SPP1*) is also known as OPN and its abnormal activation can stimulate tumor growth, invasion, angiogenesis, and immune suppression, with wide-ranging effects on cell proliferation, apoptosis, differentiation, and migration [19,20].

Previous studies [21,22] showed that the inhibition of *BCL11B *expression by siRNA selectively inhibited proliferation and effectively induced apoptosis in human T-cell acute lymphoblastic leukemia (T-ALL) cell lines (Jurkat, Molt-4). Additionally, global gene expression profiling revealed that *BCL11B *siRNA-mediated cell apoptosis may be related to BCL-2 family genes of the mitochondrial pathway, and the *TRAIL *(*TNFSF10*) gene of the death receptor signaling pathway [22], furthermore, in our previous study, the genes (*SPP1 *and *CREBBP*) of the TGF-β pathway (unpublished data). Little is known about the expression pattern of these genes in T-ALL. Thus, analyzing the expression pattern of these genes in malignant T-cells is important because *BCL11B *disruption and disturbed expression may contribute to the development of T-cell malignancies in humans [8]. In the present study, we further analyzed expression levels of *TNFSF10*, *BCL2L1*, *SPP1*, and *CREBBP*, and their correlation with *BCL11B *in male patients with T-ALL, to clarify the role of *BCL11B *in T-cell malignancies.

## Methods

### Samples

Nine newly diagnosed T-ALL patients (male, 6-28 years old; median age, 20 years; white blood cell count (WBC), 1.8-293.5 × 10^9^/L; bone marrow blast percentage: 65-93%; were recruited. The diagnosis of T-ALL was based on cytomorphology, immunohistochemistry, and cytoimmunological analysis. Peripheral blood mononuclear cells (PBMCs) from nine healthy volunteers served as controls (five males and four females, 20-45 years old; median age, 28 years). Peripheral blood was collected by heparin anticoagulation and PBMCs were separated using the Ficoll-Hypaque gradient centrifugation method. The percentage of CD3+cells in PBMCs were detected, there are 75.30 ± 26.77% (range 21.2-97.8%) in PBMCs from T-ALL samples and 59.66 ± 4.75% (range 52.4-65.8%) in PBMCs from healthy control samples.

All procedures were conducted in accordance with the guidelines of the Medical Ethics committees of the health bureau of Guangdong Province, PR China.

### RNA extraction and cDNA synthesis

RNA was extracted using the Trizol kit (Invitrogen, Carlsbad, CA, USA) and reverse transcribed into the first-strand cDNA using random hexamer primers and the reverse transcriptase Superscript II Kit (Invitrogen), according to the manufacturer's instructions.

### Real-time quantitative reverse transcription-polymerase chain reaction (qRT-PCR)

Quantitative detection of the *BCL11B *gene expression level in cDNA from PBMCs was performed using TaqMan real-time PCR. PCR was performed as described previously [8]. To precisely determine the copy numbers of *BCL11B*, a duplex vector, including a fragment of the *BCL11B *and the *β2 microglobulin *(*β2M*) genes was constructed and used as a reference (the duplex vector was a gift from Prof. C.A. Schmidt, Ernst-Moritz-Arndt University Greifswald, Germany). Based on the DNA concentration, measured by spectrophotometry and confirmed by quantitative gel eletrophoresis, standard dilutions of the vector from 10^7 ^to 10^1 ^copies were prepared [8]. Briefly, PCR was performed in a 25-μL total volume containing 2 μL of cDNA, 25 pmol of each primer (BCL11B-f and BCL11B-b for *BCL11B *gene amplification; β2Mf and β2Mb for *β2M *gene amplification), 10 nmol of each dNTP, 1.5 U AmpliTaq Gold (Applied Biosystems, Branchburg, NJ, USA), 5 pmol of 6FAM-TAMRA probe, and PCR buffer containing 4.5 mM MgCl_2_. After an initial denaturation at 95°C for 5 min, 50 cycles consisting of 95°C for 15 s and 64°C for 1 min were performed. Primers and probes for *BCL11B *and *β2M *gene amplification were synthesized by TIB Molbiol Co. (Berlin, Germany; Table 1).

**Table 1:** Sequences of primers and probes for real-time PCR (TaqMan method)

primers/probes	sequence	function
BCL11Bf	5'-CACCCCCGACGAAGATGACCAC	forward primer
BCL11Bb	5'-CGGCCCGGGCTCCAGGTAGATG	backward primer
BCL11Bp	5'-6FAM-TCACCCACGAAAGGCATCTGTCCCAAGCA-TAMRA	probe
β2Mf	5'-CTCGCGCTACTCTCTCTTTCT	forward primer
β2Mb	5'-TACATGTCTCGATCCCACTTAACTAT	backward primer
β2Mp	5'-6FAM-CTCACGTCATCCAGCAGAGAATGGAAAGTCA-TAMRA	probe

The absolute amounts of *BCL11B *and *β2M *were measured in two independent assays and *BCL11B *content per 100,000 *β2M *copies was calculated using the formula: n = 100000 × BCL11B/β2M.

Expression levels of *TNFSF10*, *BCL2L1*, *SPP1*, *CREBBP*, and the reference gene *β2-MG *were determined by SYBR Green I real-time PCR. Briefly, PCR was performed in a 25-μL total volume containing 1 μL of cDNA, 9 μL of 2.5× SYBR Green mix (Tiangen, Beijing, PR China), and 10 μmol/L primer pairs. The following cycling conditions were used: initial denaturation at 95°C for 2 min, followed by 44 cycles at 95°C for 15 s, and 81°C (*TNFSF10*, *SPP1*, *CREBBP*, and *β-2-MG*) or 84°C (*BCL2L1*) for 1 min. The relative amounts of the genes of interest and the *β2M *reference gene were measured in two independent assays. The 2^(-ΔΔCT) ^method was used to present the data of the genes of interest relative to an internal control gene [23,24]. The efficiencies of real-time PCR for expression analysis of different genes were evaluated using diluted Molt-4 cDNA (1, 5^-1^, 5^-2^, 5^-3^, 5^-4^) as templates to construct relative standard curves. Additionally, the specific amplification of PCR products was analyzed by melting curve analysis and agarose electrophoresis. Primers used in the SYBR Green I real-time PCR for all four gene amplifications were synthesized by Shanghai Biological Engineering Technology Services Co., Ltd. (Table 2).

**Table 2:** Sequences of primers for real-time PCR (SYB Green I method)

primers	sequence	function
TNFSF10	5'-GAGTATGAACAGCCCCT-3'	forward primer
TNFSF10	5'-GTTGCTTCTTCCTCTGGT-3'	backward primer
BCL2L1	5'-AAACTGGGTCGCATTGTGG-3'	forward primer
BCL2L1	5'-TCTCGGCTGCTGCATTGTTC-3'	backward primer
SPP1	5'-ACAGCCAGGACTCCATTGA-3'	forward primer
SPP1	5'-TCAGGTCTGCGAAACTTCTTAG-3'	backward primer
CREBBP	5'-CGGTTTCTCGGCGAATGAC-3'	forward primer
CREBBP	5'-CATTTCCTATTCCTGGGTTGAT-3'	backward primer

RT-PCR for *TNFSF10*, *BCL2L1*, *SPP1*, and *CREBBP *genes was performed using the same primers as described above, and the PCR products were sent to Shanghai Invitrogen Biotechnology Co. for DNA sequence analysis.

### Statistical analyses

Independent-sample *t *-test analysis was used for the *BCL11B *gene mRNA levels in different samples, while the Mann-Whitney *U *test and Spearman's rank correlation analyses were used for non-normally distributed data using the SPSS 13.0 statistical software. Differences were considered statistically significant at *P *< 0.05.

## Results

### Over-expression of *BCL11B *gene in T-ALL

**Figure 1:**
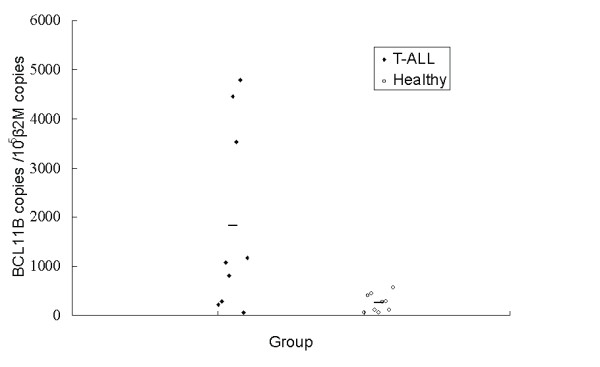
**Expression levels of the *BCL11B *gene in PBMCs from T-ALL and healthy controls**.

**Figure 2:**
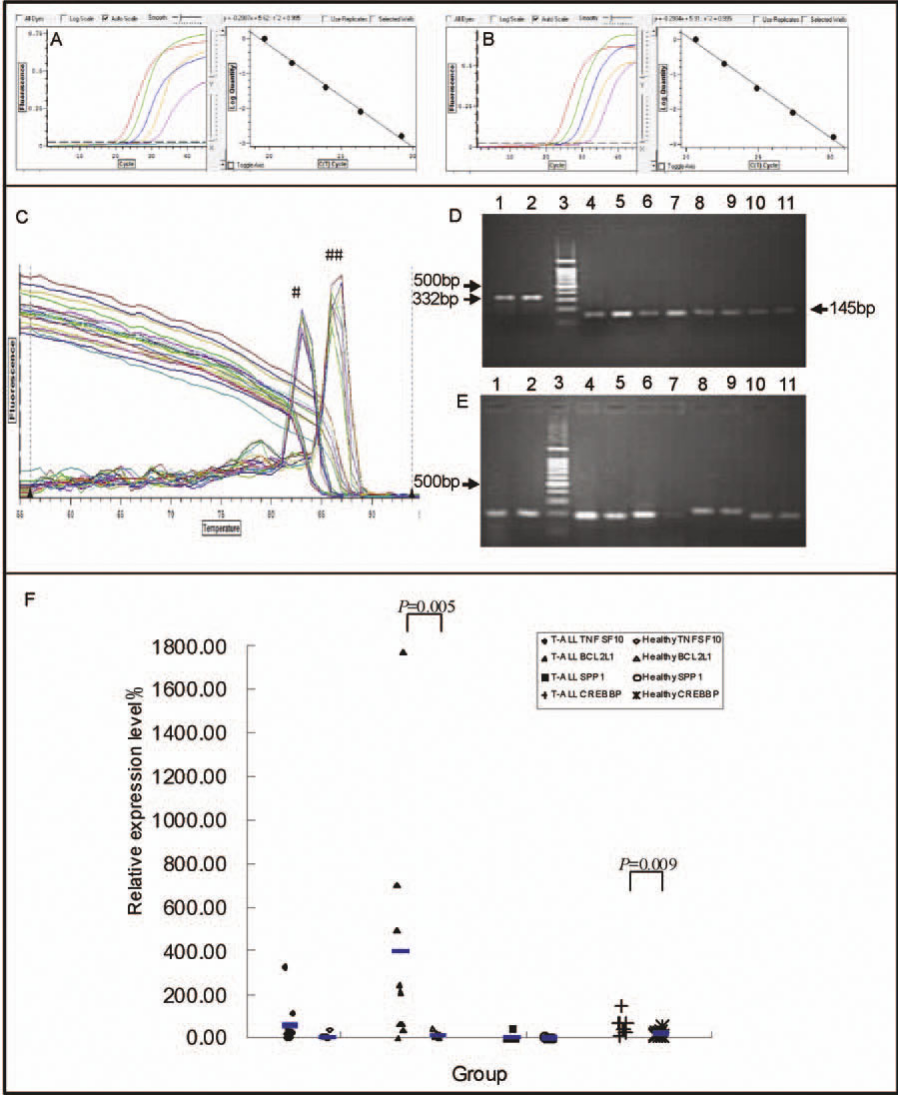
**Features of the expression of *TNFSF10*, *BCL2L1*, *SPP1*, and *CREBBP *genes in T-ALL and healthy groups**. A, B: Accurate standard curve graphs of *BCL2L1 *and the *β2M *control gene are shown using diluted Molt-4 cDNA as the template. The amplification efficiency of BCL2L1-related genes was more than 95%, and consistent with the high amplification efficiency of the *β2M *reference gene. C: Melting curves of the *BCL2L1 *and *β2M *genes from nine patients. #: Specific peak of the *β2M *reference gene begins at 81°C. ##: Specific peak of the *BCL2L1 *gene begins at 84°C. D: PCR products of the *β2M *gene by 2.5% agarose gel electrophoresis analysis. The size of the PCR products of the *β2M *gene used for the *BCL11B *reference is 332 bp (line 1, 2) and that used for the four genes of interest is 145 bp (line 4-11). Line 3: DNA ladder. E: PCR products analyzed by 2.5% agarose gel electrophoresis. Line 1-2: *BCL11B *(193bp), line 3: DNA ladder, line 4-5: *BCL2L1 *(202 bp), line 6-7: *CREBBP *(206 bp), line 8-9: *SPP1 *(241 bp), line 10-11: *TNFSF10 *(190 bp). F: Relative expression levels of the four genes of interest in T-ALL and healthy groups.

The expression level of *BCL11B *mRNA in PBMCs from patients with T-ALL (1821.81 ± 1896.58 copies/10^5 ^*β2M *copies) was significantly higher than that from healthy controls (259.71 ± 182.72 copies/10^5 ^*β2M *copies; *t *= 2.46; *P *= 0.039; Figure 1). PCR products from *β2M *and *BCL11B *genes were confirmed by 2.5% gel electrophoresis (Figure 2D, E).

### Expression of *TNFSF10*, *BCL2L1*, *SPP1*, and *CREBBP *genes in T-ALL

The high amplification efficiency of the four genes of interest (*TNFSF10*, *BCL2L1*, *SPP1*, and *CREBBP*) was consistent with that of the *β2M *reference gene. For example, the accurate standard curve graphs of *BCL2L1 *and *β_2_M *control gene amplification are illustrated in Figure 2A and 2B (r^2 ^= 0.995). The amplification efficiencies of *BCL2L1 *and the *β2M *control gene were 95.30% and 95.16%, respectively, and the melting curves are shown in Figure 2C. PCR products from the *β2M *control gene and genes of interest were confirmed using 2.5% gel electrophoresis (Figure 2D, E), followed by sequence confirmation (data not shown).

Relative expression levels of *BCL2L1 *mRNA (397.82 ± 565.98%) and *CREBBP *mRNA (53.28 ± 39.21%) in patients with T-ALL were significantly higher than those from healthy controls (*BCL2L1*: 10.83 ± 11.18%; *CREBBP*: 20.80 ± 13.50%; *P *< 0.05), whereas the relative expression levels of *TNFSF10 *and *SPP1 *mRNA showed no significant difference between T-ALL and healthy groups (Figure 2F).

**Figure 3:**
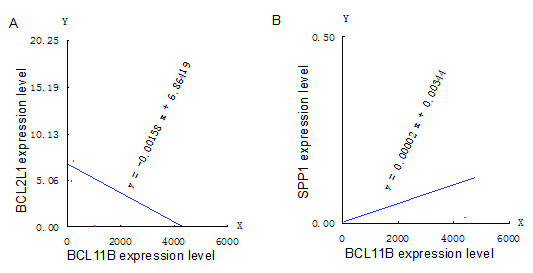
**Linear correlation analyses of the *BCL11B *and *BCL2L1 *genes (A) and *SPP1 *gene (B) in T-ALL samples**.

In T-ALL patients, Spearman's rank correlation analyses revealed that the *BCL11B *expression level was negatively correlated with the *BCL2L1 *relative expression level (*r*_s _= -0.700; *P *= 0.036; Figure 3A), and positively correlated with the *SPP1 *relative expression level (*r*_s _= 0.683; *P *= 0.042; Figure 3B). The *BCL11B *expression level did not exhibit an obvious correlation with *TNFSF10 *or *CREBBP *relative expression levels. No significant correlation was found between the *BCL11B *gene and the other four genes of interest in the healthy controls.

## Discussion

Increasing numbers of translocations involving the *BCL11B *locus [8,10,11] or high levels of *BCL11B *mRNA expression in most T-ALL cases [8,12] have been reported; however, the mechanism of *BCL11B*-mediated oncogenesis remains unknown. To clarify the role of *BCL11B *in T-cell malignancies, we further analyzed the expression levels of *TNFSF10*, *BCL2L1*, *SPP1*, and *CREBBP *genes and their correlations with *BCL11B *in patients with T-ALL and controls. Over-expression of the *BCL11B *gene, as well as *BCL2L1 *and *CREBBP *mRNA, were characteristic features of T-ALL.

Recent evidence has suggested that multiple mechanisms may regulate the release of mitochondrial factors, some of which depend on the action of caspases. *BCL2L1 *may inactivate caspase-8 by decreasing death-inducing signaling complex (DISC) formation in the plasma membrane, nucleus, and Golgi complex while diverting DISC formation to the mitochondria. The inhibitory effects of *BCL2L1 *on DISC formation may play a significant role in protecting endothelial cells from hypoxia/reoxygenation (H/R)-induced cell death [25]. Thus, over-expression of the *BCL2L1 *gene suggests that it might be related to the occurrence of T-ALL by defective regulation of apoptosis. During the process of T-ALL, over-expressed *BCL2L1 *is thought to suppress the activity of caspase-8; thus, as a kind of protection mechanism, the *TNFSF10 *gene of some patients is highly expressed, promoting caspase-8 activity in response to this abnormal cell proliferation. However, the low expression level of *SPP1 *in untreated Molt-4 cells differed from the high expression levels found in mostly solid tumors [26]. Additionally, our findings indicated no significant difference in *SPP1 *gene expression in the T-ALL group. Comprehensive analysis revealed that T-ALL occurred in the presence of *BCL11B*, *BCL2L1*, and *CREBBP *gene over-expression, which was closely related to blocking apoptosis of malignant T cell, whereas the *TNFSF10 *gene was also highly expressed in some patients, which may partly correct the imbalance.

**Figure 4:**
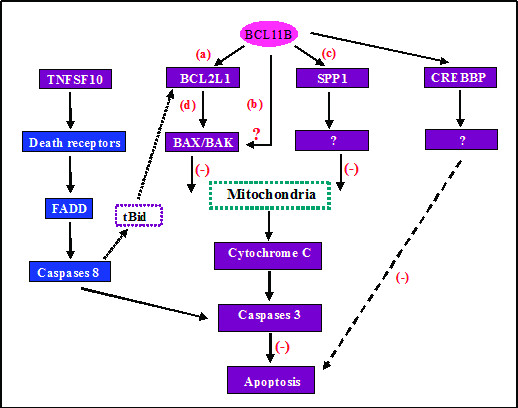
**Schematic representation of the regulatory network of apoptosis in *BCL11B *and its related genes**. (a) *BCL2L1 *is affected by the *BCL11B *gene in transcriptional regulation. (b, d) *BCL11B *and *BCL2L1 *participate in the same protein-protein interactions, along with competitive downstream target proteins. *BCL2L1 *(Bcl-xL) normally interferes with the mitochondrial programmed cell death pathway by sequestering proapoptotic proteins such as BCL2-associated × protein (BAX) and BCL2-antagonist/killer 1 (BAK1; BAK), suggesting that BAX/BAK may be competitive target proteins downstream of *BCL11B*. (c) The *SPP1 *gene may be a target of the *BCL11B *gene in transcriptional regulation: it plays a consistent role in anti-apoptotic effects with the *BCL11B *gene by decreasing mitochondrial cytochrome *c *release.

Correlation analysis of *BCL11B *in the T-ALL group revealed that the *BCL11B *expression level was negatively correlated with that of *BCL2L1 *(*Bcl-xL*), although over-expression of both genes was found in T-ALL samples. This suggested that *BCL2L1 *was affected by the *BCL11B *gene in transcriptional regulation, and both participated in the same protein-protein interactions, acting as apoptosis regulators along with a competitive target protein downstream. In *BCL11B*-knockdown T-cell lines, when exposed to growth stimuli, T cells exhibit apoptosis in S phase with concomitant decreases in the cell-cycle inhibitor p27 and the anti-apoptotic protein Bcl-xL, due to transcriptional repression [13]. However, *BCL11B *and *BCL2L1 *protein levels in the T-ALL group still remain to be validated. Correlation analysis of *BCL11B *in the T-ALL group revealed that the *BCL11B *expression level was positively correlated with the relative *SPP1 *expression level. The expression of *SPP1 *was significantly down-regulated with *BCL11B *silencing by RNA interference, suggesting that the *SPP1 *gene may be a target of the *BCL11B *gene in transcriptional regulation (unpublished data). *SPP1 *gene silencing *in vitro *significantly increased mitochondrial cytochrome *c *release, and the inhibitory action of the Wnt target gene osteopontin (*SPP1*) on mitochondrial cytochrome *c *release determines renal ischemic resistance [27]. Thus, the *SPP1 *gene may play a consistent role in anti-apoptotic effects with the *BCL11B *gene, by decreasing mitochondrial cytochrome *c *release. The hypothetical regulatory network of apoptosis in *BCL11B *and related genes is shown in Figure 4. However, the role of the *SPP1 *gene in T-cell malignancies is unclear, because low expression of *SPP1 *was detected in T-ALL.

## Conclusions

The expression pattern of the *BCL11B *gene and four of its related genes (*TNFSF10*, *BCL2L1*, *SPP1*, and *CREBBP*) was characterized in T-ALL. Over-expression of BCL11B may play a role in anti-apoptosis in T-ALL cells through up-regulation of its downstream genes *BCL2L1 *and *CREBBP*.

## Competing interests

The authors declare that they have no competing interests.

## Authors' contributions

YQL made contributions to conception and design laboratory study. XH, SHC, QS, LJY, and BL performed the laboratory technique process and the laboratory analyses. LYZ, SXG and XD were responsible of the patient's treatment and carried out acquisition of clinical data. YQL and XH coordinated the study and helped to draft the manuscript. All authors read and approved the final manuscript.
